# Gastric dysbiosis and more severe histological gastritis in symptomatic *Helicobacter pylori*-infected children and adolescents without ulcers: time to rethink early eradication?

**DOI:** 10.3389/fmicb.2026.1927606

**Published:** 2026-08-28

**Authors:** Dinka Ivulic, Camila Cabrera, Felipe Del Canto, Alejandra Gallardo, Anne Lagomarcino, Tomeu Viver, Francisco Alliende, Marcela Toledo, Gabriela Román, Francisca Jaime, Mónica González, Pamela Marchant, Marianela Rojas, Daniel Pizarro, Juan Ignacio Juanet, Mónica Villanueva, Juan Cristobal Ossa, Leandro J. Carreno, Miguel O’Ryan, Yalda Lucero

**Affiliations:** 1 Núcleo Interdisciplinario de Microbiología, Instituto de Ciencias Biomédicas (ICBM), Facultad de Medicina, Universidad de Chile, Santiago, Chile; 2 Departamento de Pediatría y Cirugía Infantil Norte, Facultad de Medicina, Universidad de Chile, Santiago, Chile; 3 Centro de Investigación Clínica Avanzada (CICA)-Hospital de Niños Roberto del Río, Facultad de Medicina, Universidad de Chile, Santiago, Chile; 4 Facultad de Medicina, Clínica Alemana de Santiago, Clínica Alemana-Universidad del Desarrollo, Santiago, Chile; 5 Dirección de Investigación, Facultad de Medicina, Universidad de Chile, Santiago, Chile; 6 Grupo de Microbiología Marina, Departamento de Diversidad Animal y Microbiana, Instituto Mediterráneo de Estudios Avanzados, Consejo Superior de Investigaciones Científicas, Universidad de las Islas Baleares, Islas Baleares, Spain; 7 Unidad de Gastroenterología Pediátrica, Hospital de Niños Roberto del Río, Santiago, Chile; 8 Unidad de Gastroenterología Pediátrica, Hospital de Niños Exequiel González Cortés, Santiago, Chile; 9 Unidad de Gastroenterología Pediátrica, Hospital Padre Hurtado, Santiago, Chile; 10 Departamento de Pediatría y Cirugía Infantil Oriente, Facultad de Medicina, Universidad de Chile, Santiago, Chile; 11 Núcleo Interdisciplinario de Farmacología e Inmunología, Instituto de Ciencias Biomédicas (ICBM), Facultad de Medicina, Universidad de Chile, Santiago, Chile

**Keywords:** 16S rRNA sequence analysis, dysbiosis, gastric microbiota, gastritis, *Helicobacter pylori*, host-pathogen interaction, pediatric gastroenterology

## Abstract

**Introduction:**

Chronic *Helicobacter pylori* (*H. pylori*) infection is a key risk factor for peptic ulcer disease (PUD) and gastric cancer in adults. However, current pediatric guidelines do not recommend eradication treatment in symptomatic children and adolescents without PUD lesions. The impact of *H. pylori* on the gastric microbiota in this population remains unclear.

**Methods:**

We conducted a prospective, multicenter study analyzing the gastric microbiota of 120 symptomatic patients aged 8–20 years undergoing endoscopy, including 41 *H. pylori-positive* and 79 *H. pylori*-negative individuals. Gastric mucosal samples were analyzed by *16S rRNA* gene sequencing. Histological gastritis severity and *H. pylori* virulence factors were also assessed.

**Results:**

*H. pylori-positive* patients exhibited a significant reduction in microbial diversity, a higher dominance index primarily due to *H. pylori*, and distinct taxonomic shifts characterized by decreased relative abundance of Firmicutes and Bacteroidota and increased Proteobacteria and Campylobacterota. Beta diversity analysis revealed significant clustering between *H. pylori-positive* and -negative groups (*p* < 0.001). No differences in microbiota composition were associated with age, pubertal stage, or gender. Histological gastritis severity correlated with specific microbiota alterations, and the presence of *H. pylori* and three co-occurring marker species distinguished severe gastritis profiles. The virulence factor CagA was associated with microbiota differences but not with histological severity.

**Discussion:**

*H. pylori* infection in symptomatic children and adolescents without PUD is associated with profound gastric dysbiosis, independent of age or pubertal status. These findings support reconsidering earlier eradication strategies to prevent persistent gastric dysbiosis and potentially reduce long-term gastric disease risk.

## 1 Introduction

*Helicobacter pylori* (*H. pylori*) is a flagellated, helical-shaped, Gram-negative bacterium that can infect the human gastric mucosa, representing the most common chronic bacterial infection globally (Malfertheiner et al., 2022; Malfertheiner et al., 2023). It is predominantly acquired at an early age within the family environment, as demonstrated in a middle-high income country such as Chile, where a prevalence of 25–30% has been described in asymptomatic school-age children and 30–40% in symptomatic children undergoing endoscopy (Cabrera et al., 2024; Lucero et al., 2021b; Serrano et al., 2017; Zabala Torrres et al., 2017). Although most individuals chronically infected with *H. pylori* are asymptomatic, some degree of histological chronic gastritis is almost universal, and peptic ulcers, intestinal metaplasia, dysplasia, and eventually gastric cancer can develop years later (Malfertheiner et al., 2023). For this reason, the Kyoto consensus defined *H. pylori*-induced gastritis as an infectious disease irrespective of clinical symptoms and complications (Sugano et al., 2015).

International guidelines recommend eradication treatment of all infected adults from countries with intermediate to high incidence of gastric cancer, regardless of symptoms and complications (Liou et al., 2025; Malfertheiner et al., 2022; Song X. et al., 2026). However, for children and adolescents, the consensus is to administer eradication treatment only in symptomatic individuals with confirmed peptic ulcer disease because the benefits of therapy in those with no peptic lesion have been controversial (Jones et al., 2017). Arguments supporting this pediatric approach include the absence of a significant lesion, the potential increase of regulatory T cell response by *H. pylori* that could have a beneficial immunological role in the host, and the detrimental effect of eradicating antimicrobial regimens on the microbiota (Jones et al., 2017). Conversely, early treatment of *H. pylori* infection could prevent the progression to ulcer development and a potential gastric cancer cascade. Since the biological processes underlying gastric carcinogenesis evolve continuously from childhood into adulthood, a strict age-based distinction between pediatric and adult management may be somewhat arbitrary, particularly when differences in regional gastric cancer risk are not considered. According to the Global Cancer Observatory (GLOBOCAN) estimates (Ervik et al., 2026), several Latin American countries, including Chile, Colombia, and Peru, have an age-standardized gastric cancer incidence (12.9–14.9 per 100,000 population) and mortality (9.2–11.4 per 100,000 population) comparable to that of Asian countries, where a “test-and-treat” strategy and eradication of infected adults have proven effective in reducing gastric cancer incidence (Malfertheiner et al., 2022; Malfertheiner et al., 2023). Therefore, whether a more proactive approach to *H. pylori* eradication should also be considered in children and adolescents living in these high-risk regions deserves further investigation.

Our group and others have reported findings strongly indicating that *H. pylori* may not be an innocuous bystander in asymptomatic children and adolescents (George et al., 2020; Lucero et al., 2021a; Lucero et al., 2021b; Zheng et al., 2025). Among *H. pylori-*positive asymptomatic children, increased plasmatic markers suggestive of gastric damage (Pepsinogen I, Pepsinogen II, and Gastrin-17) and even oncogenic markers activation have been detected (George et al., 2020; Krauthammer et al., 2026; Lucero et al., 2021a; Lucero et al., 2021b). On the other hand, alterations in gastric microbiota composition have been described comparing *H. pylori-*infected and uninfected adults and children (Ervik et al., 2026; George et al., 2020; Jones et al., 2017; Krauthammer et al., 2026; Lucero et al., 2021a; Malfertheiner et al., 2023; Zheng et al., 2025), but in the latter, most cases have been individuals with peptic ulcer disease. The literature lacks studies focusing on gastric microbiota in children and adolescents infected by *H. pylori* with non-peptic ulcer disease, the group for which eradication treatment is controversial, and the consistent finding of dysbiosis could be considered as an argument to move forward the paradigm of management to a more proactive eradication strategy.

In this study, we characterized the gastric microbiota in *H. pylori*-infected symptomatic children and adolescents without peptic ulcer disease and uninfected controls to identify specific microbiota profiles associated with histological gastritis. We hypothesized that the microbiota profile could be related to chronological age and pubertal stage. Here, we reported a dysbiotic pattern of gastric microbiota in *H. pylori-*infected subjects, associated with a higher score of histological gastritis, that was not associated with age or pubertal stage, suggesting that this dysbiotic state may be established early in life, is not harmless, and that a more proactive approach to *H. pylori* eradication during this critical developmental period should be considered to limit *H. pylori*-associated gastric dysbiosis and its potential long-term consequences.

## 2 Materials and methods

### 2.1 Ethics approval and informed consent

All protocols were approved by the Scientific Ethical Committees of the Faculty of Medicine, University of Chile (Approval No. 003-2019), and the Scientific Ethical Committees of the Faculty of Medicine, Universidad del Desarrollo–Clínica Alemana de Santiago (Approval No. 2019-39). The study protocol was also approved by the Institutional Review Board and the directors of all participating institutions. Written informed consent was obtained from all participants or their legal guardians prior to enrollment in the study.

### 2.2 Overall study design

This prospective, multicenter, observational, analytical study included individuals 8–20 years of age undergoing upper gastrointestinal endoscopy (UGIE) for dyspepsia or abdominal pain from October 2019 to January 2023 in 5 tertiary Hospitals from Santiago, Chile. Exclusion criteria included continuous use of proton pump inhibitors or histamine-2 receptor antagonists in the previous 2 weeks, antibiotic use in the past 4 weeks, peptic ulcer disease on endoscopy, active upper gastrointestinal bleeding, coagulopathy, food allergy, celiac disease, and inflammatory bowel disease. After informed consent was obtained from parents and patients 18 or older, and assent was obtained from patients under 18, a clinical standardized survey and Tanner stage evaluation were performed, followed by blood and gastric mucosa samples collection.

### 2.3 Data collection and endoscopic evaluation

A clinical standardized survey assessed demographic information, symptoms before endoscopy, familiar history of peptic ulcer disease and gastric cancer, and previous history of *H. pylori* infection and eradication. UGIE was performed following clinical protocol, and macroscopic gastric findings were classified as normal, nodular gastropathy, or congestive gastropathy.

### 2.4 Sample collection and storage

Ten milliliters of blood in EDTA tubes were obtained by puncture and stored at 4°C until processing within the first 4 h after collection.

Three mucosal gastric biopsies were obtained for histological analysis (from antrum, angle, and body) and stored in formalin until processing. One biopsy from gastric angle was obtained and immediately analyzed for rapid Urease test (DNA was also extracted from this sample and pooled with the following one); two gastric antrum biopsies were obtained for DNA and RNA extraction, respectively. The sample for RNA extraction was preserved and transported in RNA Save^®^ solution, and the DNA extraction biopsies were transported in dry ice until definitive storage at −20°C.

### 2.5 Sample processing

Tanner clinical staging performed by the pediatric gastroenterologist before the UGIE (Coleman and Coleman, 2002), in addition to plasmatic estradiol in females and testosterone in males, was used for Puberty classification. Plasma was extracted from blood samples by centrifugation at room temperature for 10 min at 200 g. Plasma estradiol levels were measured for females by radioimmune assay (Diasource, Nivelles, Belgium), and testosterone was measured for males by mass spectrometry technique. Clinical and hormonal parameters were considered for puberal stage classification; when discrepancies were detected, the parameter indicating a more advanced stage was considered.

Rapid urease test was conducted at the time of UGIE using HelicotecUT^®^ Plus (Strong Biotech Corporation, Taiwan). A color change from yellow to pink was considered positive. This gastric biopsy was subsequently frozen and used for DNA extraction.

A single-blinded pathologist performed histology. Hematoxylin and eosin-stained specimens were analyzed according to the OLGA score (Rugge et al., 2007). In addition, Giemsa-stained specimens were analyzed, and observation of *H. pylori*-like bacilli in the superficial mucosa was considered positive.

RNA extraction for *ureA* detection was performed using the RNeasy Micro^®^ kit (Qiagen, Hilden, Germany) according to the manufacturer’s instructions, and the RNA quality was assessed using electrophoresis and quantified on the Synergy™ HT instrument. RNA was reverse-transcribed to cDNA with the RevertAid RT^®^ kit (Thermo Scientific, Waltham, MA) and stored at −20°C until processing. Mic™ cycler (Bio Molecular Systems, QLD, Australia) and the SensiFAST SYBR^®^ No-ROX kit (Meridian Bioscience, London, United Kingdom) were used for real-time PCR. For the mix, 5 μl of the master mix, 2 μL of water, 400 nM of each primer (F-CGTGGCAAGCATGATCCAT and R-GGGTATGCACGGTTACGAGTTT), and 2 μL of cDNA diluted 1:10 in water were used for a final volume of 10 μL. The program used an annealing temperature of 60°C. For *ureA*, amplifying the curve at any Ct accompanied by a melting curve of 82°C was considered positive.

Three tests were used to define positivity for *H. pylori* infection: (1) rapid urease test; (2) identification of bacilli associated with gastric epithelia on Giemsa-stained biopsies; (3) RT-PCR of the *ureA* gene. A subject was considered positive if *H. pylori* was detected by at least two of these assays.

DNA extraction for microbiota analysis, *cagA*, and *vacA* gene detection was performed using the QIAmp^®^ DNA Micro kit (Qiagen, Hilden, Germany), and DNA quality was assessed using the electrophoresis technique and quantified on the Synergy™ HT instrument. DNA was aliquoted and stored at −80°C until processing. *cagA* were assessed by a nested PCR, with external primers in a conventional PCR at the first step and then internal primers in real-time PCR as the second step, as described by Wormwood et al. previously (Wormwood et al., 2018). An amplification of the curve at any Ct accompanied by a melting curve of 80°C was considered positive. For *vacA*, two PCR analyses were performed for the s1/s2 and m1/m2 alleles separately according to the protocols described by Rudi et al. (1998). The success of the amplification was checked by electrophoresis in a 2% agarose gel with a 290 and 352 pb band for m1 and m2, respectively.

### 2.6 Microbiota gene amplification and sequencing process

Primers 341F (CCTACGGGNGGCWGCAG) and 799R (CMGGGTATCTAATCCKGTT) were used to amplify the 16S rRNA gene V3–V4 variable region. A 30–35 cycle PCR was performed using the HotStarTaq Plus Master Mix Kit (Qiagen, United States) with an annealing temperature of 53°C. The success of the amplification was checked by electrophoresis in a 2% agarose gel. Then, purified PCR products by calibrated Ampure XP beads were used to prepare the Illumina DNA library. Two process control samples without a piece of tissue were included. Finally, the sequencing procedure was performed at MR DNA Shallowater, TX, United States)^1^ on an Illumina MiSeq platform following the manufacturer’s guidelines.

### 2.7 Microbiota amplicon sequence variants (ASV) clustering and operational phylogenetic unit (OPU) assignment

Raw sequencing data was analyzed using the Qiime2 bioinformatics platform (Bokulich et al., 2018) with the parameters –p-trunc-len-f 280, –p-trunc-len-r 220, –p-trim-left-f 19 and –p-trim-left-r 22. ASVs were performed with DADA2 software implemented in Qiime2. The longest sequence of each ASV was selected as representative, and the bacterial and archaeal ASVs sequences were aligned using the non-redundant SILVA REF 138.1 database as a reference (Quast et al., 2013) implemented in the ARB package (Ludwig et al., 2004), and the alignment was performed using the SINA tool (Pruesse et al., 2012).

Taxonomic assignment was performed by phylogenetic inference. The aligned ASV representative sequences were included in the preexisting non-redundant SILVA REF 138.1 phylogenetic tree using the parsimony tool to pick the closest relatives and generate a phylogenetic tree using the neighbor-joining method. Finally, the tree was manually curated to obtain the assigned OPUs. OPUs with mitochondrial or chloroplast assignation were removed. An OPU was considered the smallest monophyletic clade grouping ASV representatives and their closest reference sequence. Where achievable, OPUs comprised a type strain as a reference sequence. If not possible, other available databases were consulted. Reads were considered to belong to the same species when the identity values were > 98.7% when aligned to the representative strain sequence (Stackebrandt and Ebers, 2006). Reads were supposed to be a different unclassified species of the same genus when the identity percentage with the closest relative type strain was between 94.5 and 98.7% (Stackebrandt and Ebers, 2006).

Indices of alpha diversity for each sample and rarefaction curves were calculated using the PAST software v4.13 over the total prokaryotic reads (Hammer et al., 2001). OPUs harboring < 2 sequences and those occurring in a single sample were removed for the rest of the comparative data analyses. Data scaling was performed using the total sum scaling (TSS) method, transforming reads into relative abundances by dividing the number of reads from each OPU by the total number of reads within the sample. Comparative analyses (HeatMap, PCoA, HeatTree, Random Forest, LDA- LEfSe, and correlations network) of microbiome data were performed using MicrobiomeAnalyst 2.0 (Lu et al., 2023). Detailed options and selected parameters for each analysis can be found in the figure legends. For the oxygen requirement analysis, each OPU was manually classified according to its oxygen requirement (aerobic, anaerobic, facultatively anaerobic, or microaerophilic) using information from the original species descriptions, the BacDive database (Reimer et al., 2022), and the MiDAS database (Dueholm et al., 2023). Sankey diagrams were generated using SankeyMATIC.^2^

### 2.8 Data management and analysis

The REDCap^®^ platform was used to store demographical, clinical, and endoscopic data and the results obtained from the laboratory techniques. NorayBanks Plus^®^ was used to register sample storage and logistics according to Biobank standards.

Statistical analyses were performed using R software (version 4.2.3; R Core Team, Vienna, Austria). Data normality and homoscedasticity were assessed before group comparisons. Since age was not normally distributed, groups were compared using the Mann-Whitney U test. Categorical variables were compared using the Chi-square or Fisher’s exact test, depending on the expected cell frequencies, and odds ratios (ORs) with 95% confidence intervals (95% CI) were calculated when appropriate.

Microbiome alpha-diversity indices were compared using the Wilcoxon rank-sum test. Beta diversity was evaluated using Bray-Curtis dissimilarity and visualized by principal coordinate analysis (PCoA), with group differences assessed by permutational multivariate analysis of variance (PERMANOVA). Differentially abundant taxa were explored using Heat Tree analysis and Random Forest classification. Taxa associated with histological gastritis severity were further identified using linear discriminant analysis effect size (LEfSe), applying false discovery rate (FDR)-adjusted Kruskal-Wallis tests. Correlation networks were inferred using SparCC. Unless otherwise specified, all statistical tests were two-sided, and statistical significance was defined as *p* < 0.05.

## 3 Results

### 3.1 Subjects, samples, sequences, and OPU distribution

**FIGURE 1:**
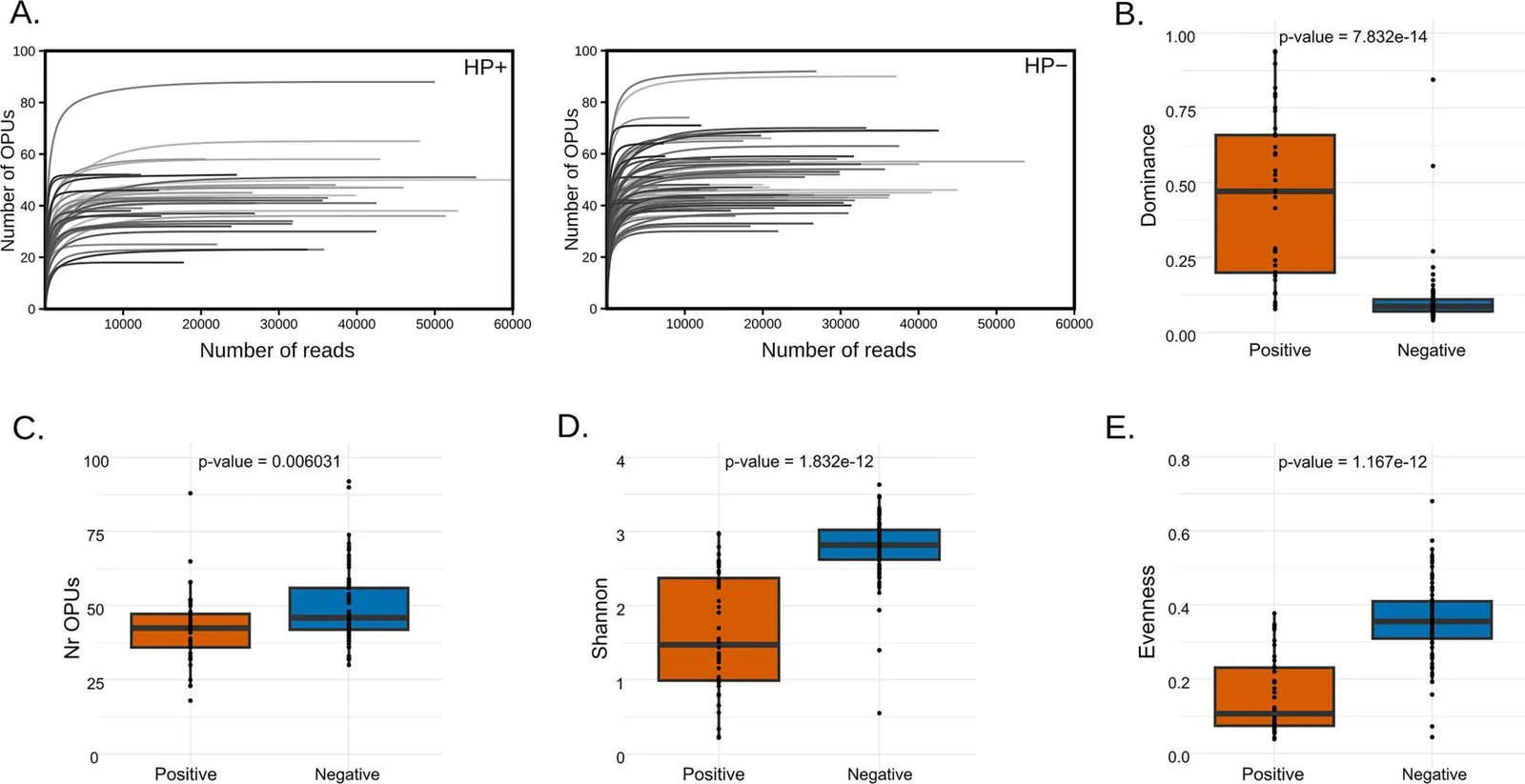
Rarefaction and alpha diversity indices according to *H. pylori* infection status. **(A)** Rarefaction curves for detected OPUs, where each line represents an independent sample. Box plots for **(B)** Dominance, **(C)** number of OPUs, **(D)** Shannon-Weiner index, and **(E)** Evenness were plotted for each *H. pylori* group. The Wilcoxon rank sum test was used to compare differences (*p* < 0.05) between groups.

Forty one *H. pylori-*positive and 79 *H. pylori-*negative individuals were recruited. Gender, chronological age, and pubertal stage distribution were similar in both groups, as well as family history of significant gastric disease and clinical presentation (Table 1). The *H. pylori-*positive group had a higher frequency of nodular gastritis and a significantly higher severity of histological gastritis (Figure 1A and Table 1).

**TABLE 1:** Demographic and clinical characteristics of the patients according to *H. pylori* status of infection.

Characteristics		*H. pylori*-negative	*H. pylori-positive*	Total	OR	*P*
Total		79	41	120		
Female, n (%)		59 (74.7)	24 (58.5)	83 (69.2)	0.482 (0.214–1.08)	0.069
Age in years, median (IQ range)		14 (8–20)	14 (8–20)	14 (8–20)		NS
Pubertal Stage, n (%)	Tanner I	8 (10.1)	3 (7.3)	11 (9.2)		NS
	Tanner II	4 (5.1)	5 (12.2)	9 (7.5)		
	Tanner III	20 (25.3)	9 (22.0)	29 (24.2)		
	Tanner IV	22 (27.8)	12 (29.3)	34 (28.3)		
	Tanner V	25 (31.6)	12 (29.3)	37 (30.1)		
Symptoms prompting endoscopy, n (%)*	Epigastric pain	53 (6)	34 (82.9)	87 (74.4)	2.070 (0.826–5.775)	NS
	Nocturnal abdominal pain	36 (46.8)	20 (48.8)	56 (47.5)	1.084 (0.503–2.333)	NS
	Melena	10 (13.0)	3 (7.3)	13 (11.3)	0.549 (0.112–1.963)	NS
	Persistent vomiting	14 (18.2)	10 (24.4)	24 (20.6)	1.550 (0.597-3.925)	NS
	Hematemesis	4 (5.2)	3 (7.3)	7 (6.0)	1.491 (0.263–7.491)	NS
	Weight loss	31 (40.3)	12 (29.3)	43 (37.1)	0.628 (0.269–1.408)	NS
	Loss of appetite	37 (48.1)	24 (58.5)	61 (51.7)	1.517 (0.705–3.318)	NS
	Anemia	11 (14.3)	3 (7.3)	14 (12.2)	0.512 (0.105–1.799)	NS
Family history, n (%)^†^	Gastric cancer	12 (15.4)	11 (26.8)	23 (19.2)	2.005 (0.779-5.143)	NS
	Duodenal ulcers	18 (23.1)	7 (17.1)	25 (20.8)	0.684 (0.241–1.763)	NS
	Gastritis	45 (57.7)	26 (63.4)	71 (59.2)	1.265 (0.582–2.812)	NS
Gastric endoscopic finding, n (%)	Normal	43 (54.4)	4 (9.8)	47 (39.2)	10.52 (3.75–38.68)	<0.001
	Nodular gastropathy	3 (3.8)	20 (48.8)	23 (19.2)		
	Congestive gastropathy	33 (41.8)	17 (41.5)	50 (41.7)		
Gastric histological findings, n (%)	Gastritis	58 (73.4)	40 (97.6)	97 (80.8)	13.45 (2.65–329.37)	0.001
	No inflammation	21 (26.6)	1 (2.4)	22 (18.3)	3.72 (0.293–119.295)	0.001
	Mild inflammation	55 (69.6)	7 (17.1)	62 (51.7)		
	Moderate inflammation	3 (3.8)	31 (75.6)	34 (28.3)		
	Severe inflammation	0 (0)	2 (4.9)	2 (1.7)		
	No atrophy	78 (98.7)	39 (95.1)	117 (97.5)		NS
	Mucosal atrophy	1 (1.3)	2 (4.9)	3 (2.5)		
	No metaplasia	76 (96.2)	41 (100)	117 (97.5)		NS
	Intestinal metaplasia	3 (3.8)	0 (0)	3 (2.5)		

*Percentages were calculated using 77 participants with available data in the *H. pylori*-negative group; no missing data occurred in the *H. pylori*-positive group.

† Percentages were calculated using 78 participants with available data in the *H. pylori*-negative group; no missing data occurred in the *H. pylori*-positive group.

Gastric microbiota could be assessed in 118 individuals (40 *H. pylori-*positive and 78 *H. pylori*-negative). A total of 2,983,607 prokaryotic reads were obtained, with a median of 29,533 sequences for the *H. pylori-*positive group and a median of 21,143 sequences for the *H. pylori*-negative group. These sequences were grouped into ASV followed by OPUs, obtaining a total of 462 different OPUs according to phylogenetic inference, with a median of 43 OPUs for *H. pylori-*positive and 46 OPUs for *H. pylori*-negative samples (Supplementary Table 1). After discarding OPUs with < 2 sequences or present in a single sample, as they could be considered artifacts, the data set was reduced from 462 to 293 total OPUs. Eleven OPUs for Archaea and 282 OPUs for Bacteria were identified.

### 3.2 Alpha diversity is affected by the presence of *H. pylori*

The number of species and their distribution were first analyzed for alpha diversity indices. Figure 1A shows rarefaction curves for the number of reads and OPUs analyzed. All samples reached the sequencing plateau, indicating that the sequencing depth was optimal and that most diversity was recovered. This agrees with the Chao-1 alpha diversity index results, which represent the number of estimated OPUs, and the results of the number of observed OPUs (Supplementary Table 1), which coincide in all samples, indicating again that the optimal sequencing depth was reached in the libraries.

When the *H. pylori-positive* and *H. pylori*-negative groups were compared, differences were observed in all the alpha diversity indices analyzed. A higher dominance index was observed in *H. pylori-positive*, which is mainly attributed to the presence of *H. pylori* (Supplementary Figure 1), indicating that this taxon predominates by far in the *H. pylori-positive* community (Figure 1B). This agrees with a significantly lower number of OPUs (Figure 1C) and a substantially lower heterogeneity shown in the Shannon-Weiner index (Figure 1D). Furthermore, a lower Evenness was observed in the *H. pylori-positive* group (Figure 1E), which describes the distribution of abundance across the species in a community (Moore, 2013).

### 3.3 The gastric microbiota differs between *H. pylori*-positive and *H. pylori*-negative subjects

**FIGURE 2:**
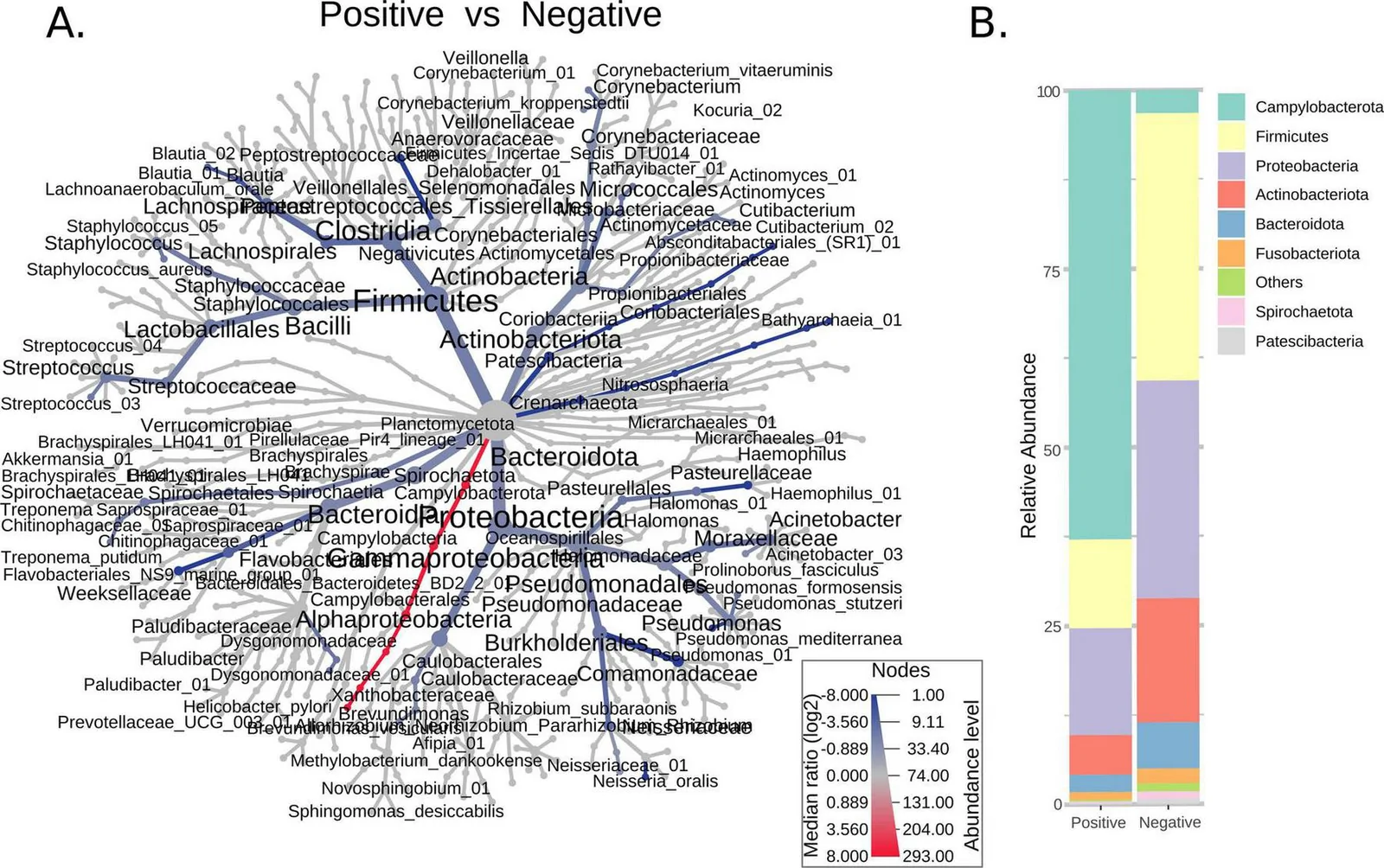
Heat Tree and relative abundance analysis according to *H. pylori* infection status. **(A)** Heat tree that depicts the taxonomic differences between *H. pylori-positive* and *H. pylori*-negative groups, up to the species level. Only significant taxon names are colored, and colors indicate whether corresponding taxa are lower (blue) or higher (red) in *H. pylori-positive* compared to *H. pylori*-negative. The median abundance and the statistical non-parametric Wilcoxon rank sum test (*p* < 0.05) were used. **(B)** Relative abundances (%) bar plot of the top 8 most frequent phyla. The rest phyla were represented as “Others.”

A Heat Tree was built to compare the differences between *H. pylori-positive* and *H. pylori*-negative samples to find differential characteristics within each taxonomic level (Foster et al., 2017; Figure 2A). Ten phyla, 19 classes, 26 orders, 34 families, 35 genera, and 47 species showed nominally significant differences (Wilcoxon rank-sum test, *p* < 0.05) in the Heat Tree. After Benjamini-Hochberg FDR correction within each taxonomic level, 9 phyla, 14 classes, 19 orders, 17 families, 15 genera, and 14 species remained significant (*q* < 0.05) (Supplementary Table 2). At the phylum level, Campylobacterota, Firmicutes, Proteobacteria, Actinobacteriota, and Bacteroidota stand out for being statistically different (FDR) and for their contribution to the changes in relative abundance between *H. pylori-positive* and *H. pylori*-negative (Figure 2B).

We observed an increase in Campylobacterota at the expense of the other four phyla in the *H. pylori-positive* group. Within the Campylobacterota phylum, *H. pylori* was the only species with a significantly different abundance, explaining the main changes between both groups (Supplementary Figure 1). In the case of the Firmicutes phylum, *Streptococcus_03* stands out for having statistically different abundance and for its contribution within the primary 20 species when comparing both groups (Supplementary Figure 2 and Supplementary Table 2). Likewise, *Pseudomonas formosensis* and *Brevundimonas vesicularis* are the major species of the phylum Proteobacteria with statistically different abundances between groups. Next, *Corynebacterium kroppenstedtii* was the main species belonging to the phylum Actinobacteriota, with statistically different abundance between *H. pylori*-positive and *H. pylori*-negative groups. Finally, within the Bacteroidota phylum, an unidentified species from the Chitinophagaceae family was the most relevant (Supplementary Table 2).

**FIGURE 3:**
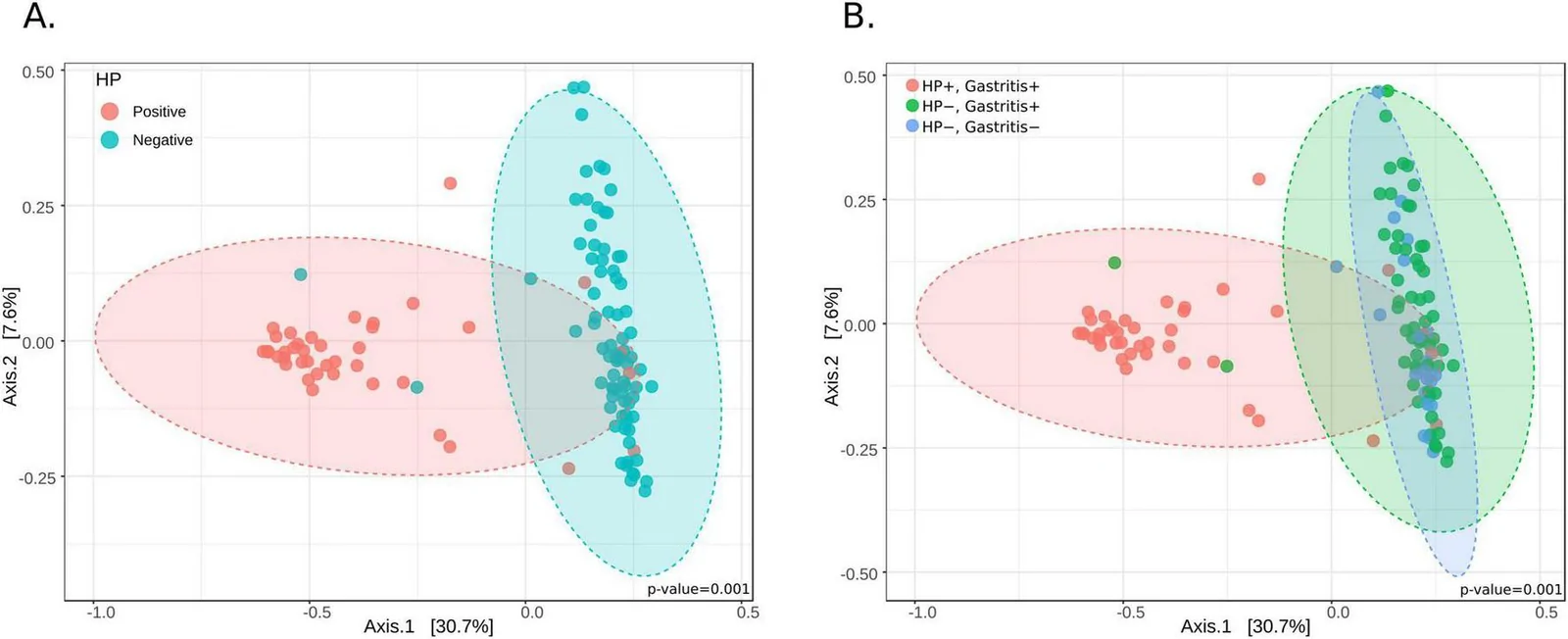
Beta diversity analysis according to *H. pylori* infection status. **(A)** Comparison at OPU level between *H. pylori*-positive and *H. pylori*-negative samples. **(B)** Comparison at OPU level between *H. pylori*-positive and -negative groups considering the presence of gastritis. Analysis performed using Bray-Curtis measures of beta diversity and PERMANOVA statistics. HP^+^: *H. pylori*-positive group; HP^–^: *H. pylori*-negative group.

To determine whether the overall composition of the mucosa-associated gastric microbiota differed according to *H. pylori* infection status, beta diversity was assessed using principal coordinate analysis (PCoA) based on Bray-Curtis dissimilarity (Figure 3A). The first two principal coordinates explained 38.3% of the total variation (Axis 1: 30.7%; Axis 2: 7.6%). PERMANOVA demonstrated a significant difference in overall microbial community composition between *H. pylori*-positive and *H. pylori*-negative subjects (*p* = 0.001). Group separation was primarily driven by differences along Axis 1 (Wilcoxon rank-sum test, *p* < 0.001), whereas Axis 2 showed no significant between-group differences. These findings indicate the presence of two distinct gastric microbiota profiles associated with *H. pylori* infection status.

To investigate whether the presence or absence of histological gastritis was associated with differences in the microbiota, a beta diversity analysis was performed, considering the presence or absence of this condition. All samples classified as *H. pylori-positive* had the presence of gastritis (*n* = 40), while samples classified as *H. pylori*-negative were divided between those with histological gastritis (*n* = 57) and those with normal histology (*n* = 21). No difference was observed between the “*H. pylori*-negative, gastritis+” and the “*H. pylori*-negative, gastritis–” groups (*p* = 0.573), but significant differences were observed between the “*H. pylori-positive*, gastritis+” and both *H. pylori*-negative groups with and without gastritis (*p* = 0.001) (Figure 3B).

**FIGURE 4:**
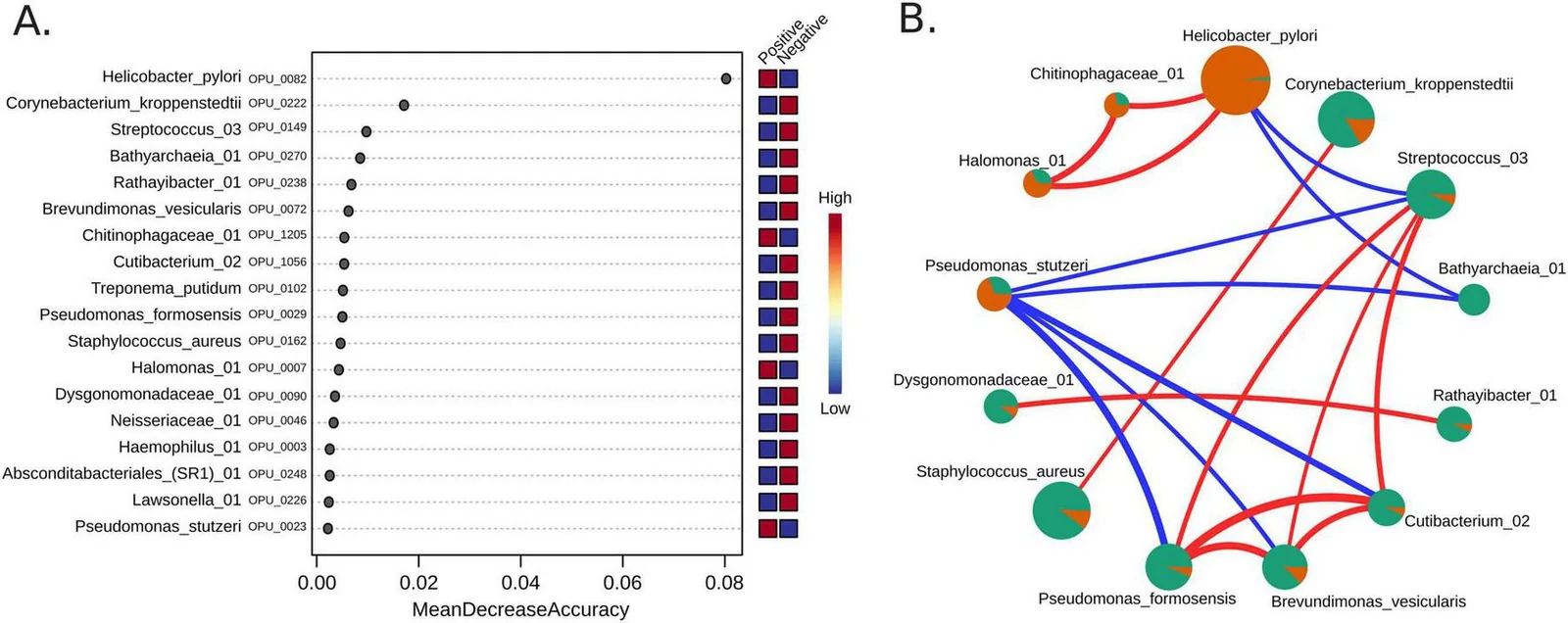
Random Forest and correlation network analysis according to *H. pylori* infection status. **(A)** Top 18 bacteria to discriminate between *H. pylori*-positive and -negative groups predicted by the Random Forest algorithm (ntree = 5,000; mtry = 17). **(B)** Correlation network (SparCC) of 18 species depicted on the Random Forest. Correlation threshold > 0.3 and *p*-value threshold < 0.05 were used. The nodes (pie charts) represent species, size reflects relative median abundance. Positive and negative correlations in red and blue lines, with line thickness representing the strength of correlation. Values of correlations are given in Supplementary Table 3.

Therefore, the differences observed in the Heat Tree could explain the different composition revealed by the beta diversity analysis on the *H. pylori-positive* group. A Random Forest analysis was performed to delve deeper into identifying significant features or potential biomarkers in microbiota species other than *H. pylori*. Figure 4A shows the top 18 species identified as marker species between the *H. pylori-posivite* and *H. pylori*-negative groups. A total of 4 marker species were found for the *H. pylori-positive* group: *H. pylori*, an unidentified species of the Chitinophagaceae family, an unidentified species of the *Halomonas* genus, and *Pseudomonas stutzeri*. In contrast, 14 species best represented the HP– group: *Corynebacterium kroppenstedtii*, an unidentified species of the *Streptococcus* genus, an unidentified species of the Bathyarchaeia class, an unidentified species of the *Rathayibacter* genus, *Brevundimonas vesicularis*, an unidentified species of the *Cutibacterium* genus, *Treponema putidum*, *Pseudomonas formosensis*, *Staphylococcus aureus*, an unidentified species of the Dysgonomonadaceae family, an unidentified species of the Neisseriaceae family, an unidentified species of the *Haemophilus* genus, an unidentified species of the Absconditabacteriales order and, an unidentified species of the *Lawsonella* genus.

To discover possible bacterial interactions that could help understand how the microbiota responds and adapts to *H. pylori* infection, a correlation network was built on the 18 species of the Random Forest. A network with 13 species and 18 connections was observed with a cutoff of *r* = 0.3 (Figure 4B). The highest positive correlations ( > 0.5) were those observed between the following pairs (Supplementary Table 3): *Cutibacterium*_02/*Pseudomonas formosensis* (0.684), *Brevundimonas vesicularis*/*Pseudomonas formosensis* (0.596), *Brevundimonas vesicularis*/*Cutibacterium*_02 (0.523), *Chitinophagaceae*_01/*Halomonas*_01 (0.512). On the other hand, the most significant negative correlations ( < −0.5) were between pairs *Pseudomonas formosensis*/*Pseudomonas stutzeri* (−0.586) and *Cutibacterium_02*/ *Pseudomonas stutzeri* (−0.518). Only positive correlations were observed within the four species classified as *H. pylori-positive* and within the nine species classified as *H. pylori*-negative. Interestingly, *H. pylori* and *Pseudomonas stutzeri* were the only negatively correlated species in the *H. pylori*-negative group.

### 3.4 Gastric microbiota composition was not associated with age, pubertal stage, and gender

**FIGURE 5:**
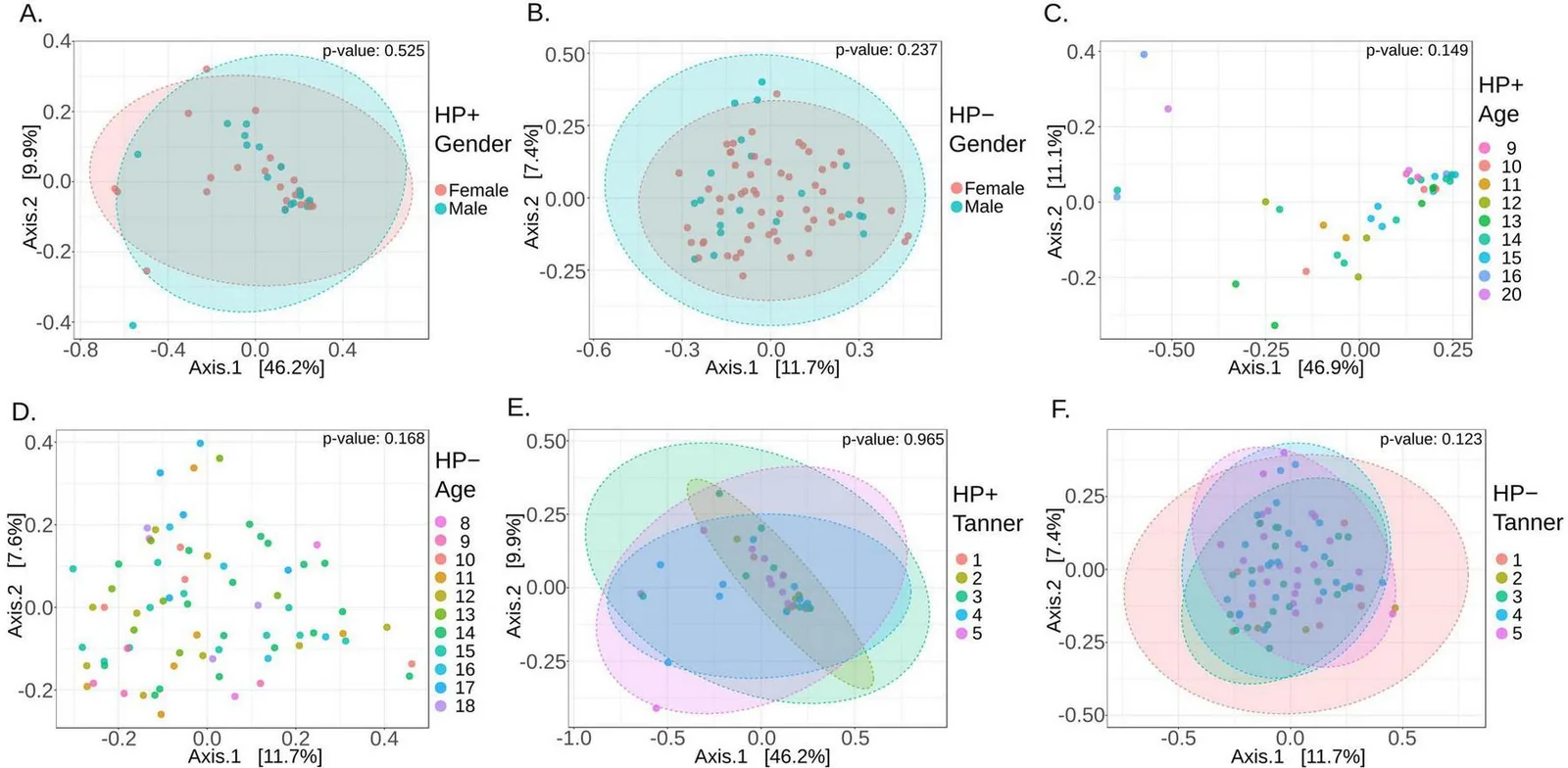
Beta diversity analysis according to age, pubertal stage, and gender among HP groups. PCoA plot for **(A)** *H. pylori-positive* Gender, **(B)** *H. pylori*-negative Gender, **(C)** *H. pylori-positive* Age, **(D)** *H. pylori*-negative Age, **(E)** *H. pylori-positive* Final Tanner, **(F)** *H. pylori*-negative Final Tanner. Analysis performed using Bray–Curtis measures of beta diversity and PERMANOVA statistics. HP^+^: *H. pylori*-positive group; HP^–^: *H. pylori*-negative group.

Considering the different immunological responses against *H. pylori* infection described in children and adults that impact different therapeutic approaches, we were interested in determining if age and pubertal stage influence the gastric microbiota composition. No differences were found by PCoA analysis when analyzing age or pubertal status by separating *H. pylori-positive* and *H. pylori*-negative groups (Figures 5A,B,D,E). On the other hand, considering that during pubertal development, sexual hormones could also impact immunological responses against infections, we explored the association between gastric microbiota composition and gender. The composition of the gastric microbiota did not differ between genders in either *H. pylori-positive* (Figure 5C) or *H. pylori*-negative (Figure 5E) groups. When the data were analyzed without separation by the detection of *H. pylori*, the same results were observed, concluding that there were no differences in the composition of the microbiota associated with age, pubertal stage, and gender.

### 3.5 Relationship between histological severity, *H. pylori* virulence factors, and the gastric microbiota

**FIGURE 6:**
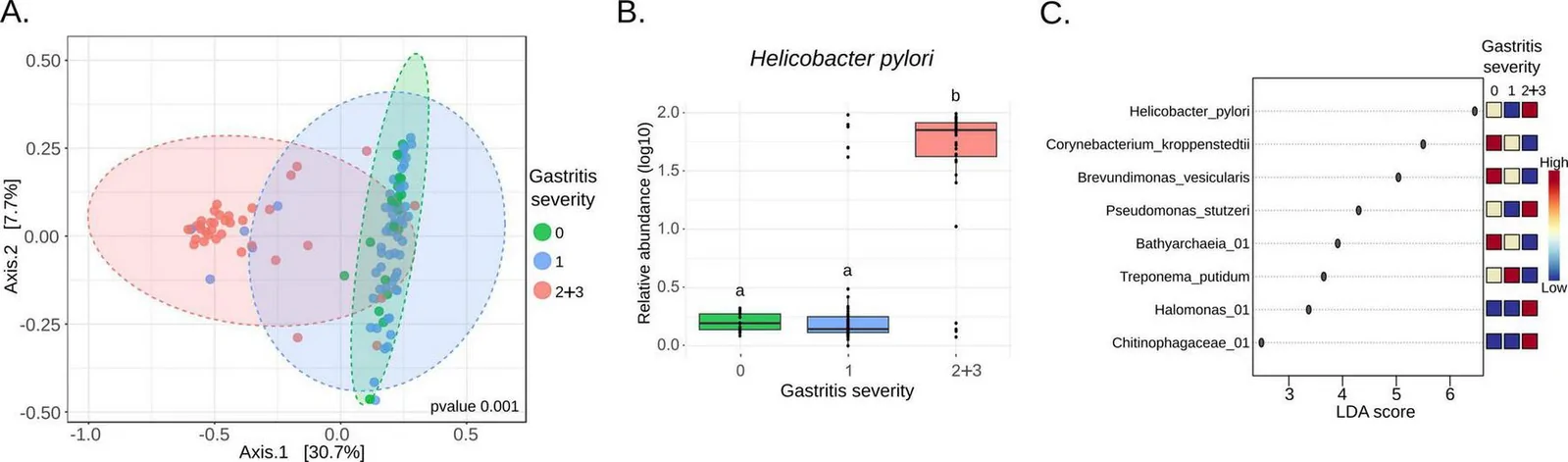
Beta diversity analysis according to severity of histological gastritis, that was coded into four groups: severity “0” (no gastritis, *n* = 20), severity “1” (mild gastritis, *n* = 61), severity “2” (moderate gastritis, *n* = 34), severity “3” (severe gastritis, *n* = 2). **(A)** Gastritis severity PCoA analysis using Bray–Curtis measures of beta diversity. Statistics: PERMANOVA. **(B)** *H. pylori* relative abundances (Log10) comparison between groups according to gastritis severity. Statistic Kruskal-Wallis and Bonferroni’s *post hoc*. Different letters mean differences between groups (*p* < 0.05). **(C)** LEfSe-LDA score 2 for identification of species that differ significantly in abundance between groups (*p*-value cutoff < 0.05 FDR-adjusted, Kruskal–Wallis test).

Next, we focused on evaluating how the severity of gastritis is related to the composition of the microbiota. For this, we coded the severity of histological gastritis into three groups: “severity 0” (no gastritis, *n* = 20), “severity 1” (mild gastritis, *n* = 61), and “severity 2+3” (moderate gastritis, *n* = 34 + severe gastritis, *n* = 2). When performing PCoA analysis, the microbiota composition was different (Figure 6A). By pairwise comparison, differences between “severity 0” and “severity 2+3” (*p* = 0.002), and between “severity 1” and “2+3” (*p* = 0.002), and not between severity category “0” and “1” (*p* = 0.462) were observed. Furthermore, we were able to determine differences in the relative abundance of *H. pylori* between the histological severity groups (Figure 6B). Thus, the relative abundance of *H. pylori* was different between categories “0” and “2+3,” as well as between categories “1” and “2+3,” but it was not different between categories “0” and “1.” To explore the identification of significant features or potential biomarkers related to histological gastritis severity, a linear discriminant analysis effect size (LEfSe) was performed (Figure 6C). We identified eight species differing between the severity categories “0,” “1” and “2+3.” Four species best represented the severity “2+3” group, which coincides with those of the *H. pylori-positive* group in Figure 4A. The remaining four, associated with severity “0” and “1,” also coincide with those represented as the *H. pylori*-negative group marker species.

**FIGURE 7:**
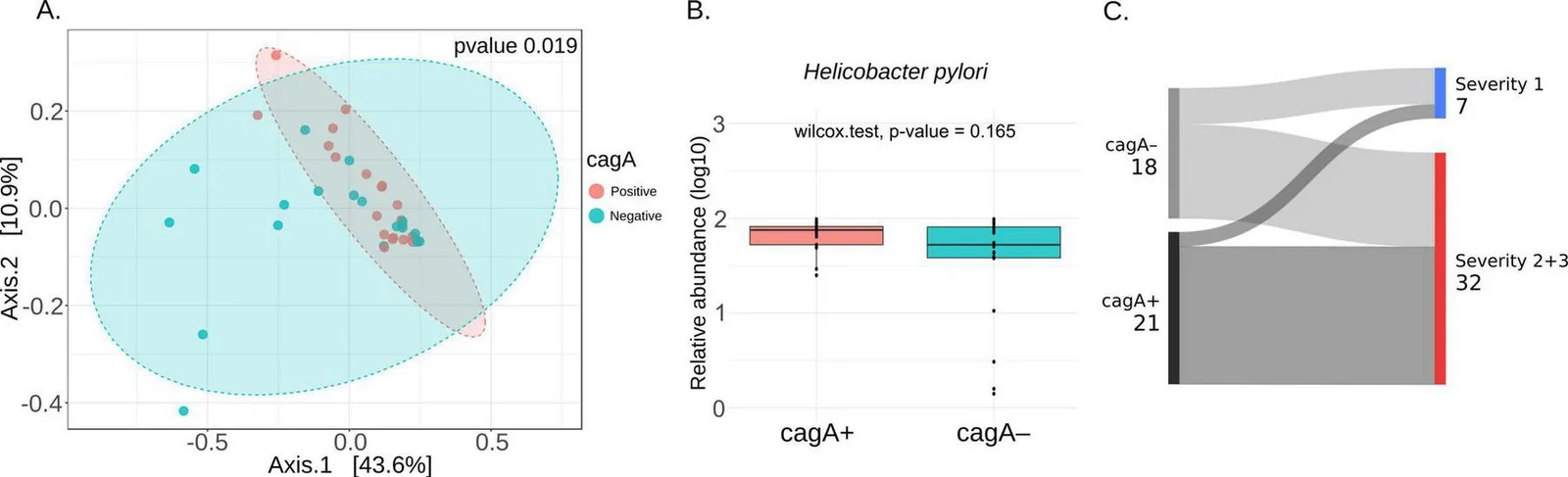
Beta diversity analysis according to the presence of *cagA* virulence factor in *H. pylori* infected subjects and its relationship with severity of histological gastritis. Two groups of *H. pylori-positive* patients were formed: *cagA* positive (*n* = 22), and *cagA* negative (*n* = 18). **(A)** PCoA analysis using Bray–Curtis measures of beta diversity. Statistics: PERMANOVA. **(B)** *H. pylori* relative abundances (log10) comparison between groups. The Wilcoxon rank sum test was used to compare differences (*p* < 0.05) between groups. **(C)** Sankey plot shows the number of samples connecting from “*cagA*” to “Gastritis severity.”

**FIGURE 8:**
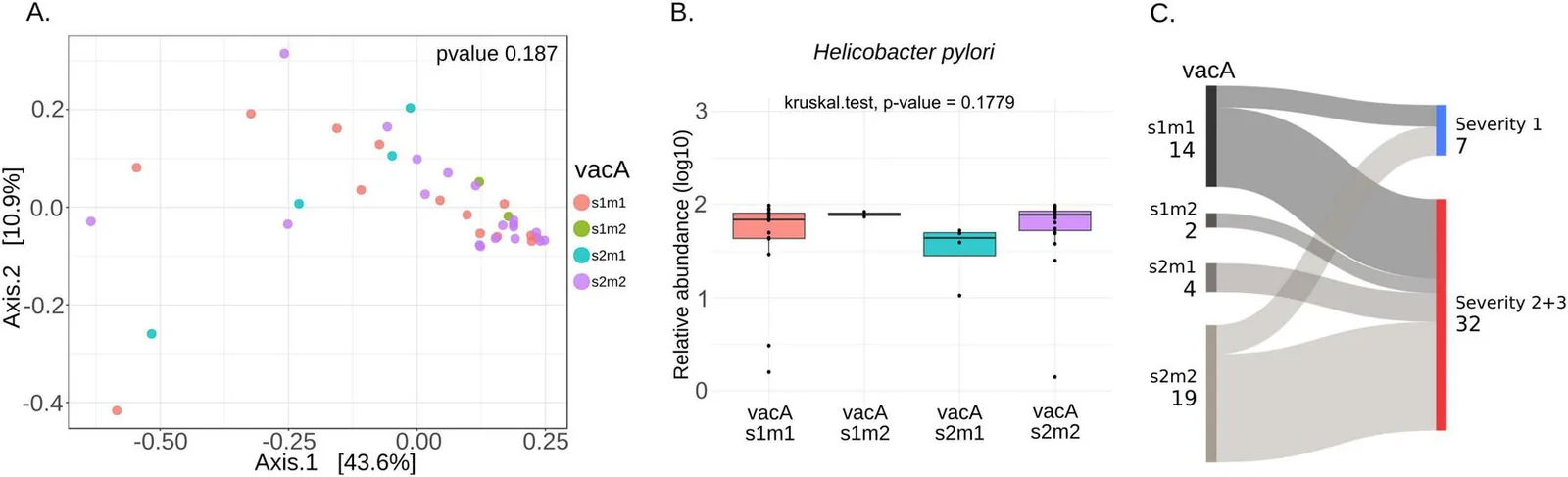
Beta diversity analysis according to the genotype of *vacA* virulence factor in *H. pylori* infected subjects and its relationship with severity of histological gastritis. Within the *H. pylori-positive* group, four groups were formed: *vacA* s1m1 (*n* = 14), *vacA* s1m2 (*n* = 2), *vacA* s2m1 (*n* = 4), and *vacA* s2m2 (*n* = 19). **(A)** PCoA analysis using Bray–Curtis measures of beta diversity. Statistics: PERMANOVA. **(B)** *H. pylori* relative abundances (log10) comparison between groups. Statistic Kruskal-Wallis and Bonferroni’s *post hoc*. **(C)** Sankey plot shows the number of samples connecting from “vacA” to “Gastritis severity.”

Analyzing the potential effect of *H. pylori* virulence factors on the composition of the gastric microbiota and its relationship with the severity of gastritis, we included 22 *cagA*+ samples (55%) and 18 *cagA*- samples, observing a difference in the PCoA analysis (*p* = 0.019) (Figure 7A). When searching for potential bacterial markers, no significant features (OPUs) were identified in the LEfSe analysis (FDR-adjusted 0.05, LefSe-LDA score 2). The participation of the relative abundance of *H. pylori* had no effect (Figure 7B). As seen in the Sankey diagram (Figure 7C), no significant association was evidenced between the presence of *cagA* and histological severity (*p* = 0.211, Fisher exact test). Similar differences were not detected for *vacA* virulence factor (*p* = 0.187) (Figure 8A). The participation of the relative abundance of *H. pylori* was not significantly different between groups (Figure 8B), and histological gastritis severity does not appear to be strongly associated with any of the *vacA* variants (Figure 8C).

### 3.6 Taxonomically inferred metabolic and physiological characteristics of the gastric microbiota according to *Helicobacter pylori* infection status.

The metabolic and physiological characteristics of the 18 marker taxa identified by the Random Forest analysis (Figure 4A) were inferred from published species descriptions and curated reference databases (Table 2). In the *H. pylori-positive* group, all marker taxa were Gram-negative bacteria, whereas the *H. pylori*-negative group included Gram-negative bacteria, Gram-positive bacteria, and one archaeal taxon (*Bathyarchaea*_01). Regarding urease activity, *H. pylori* and, based on available taxonomic information, *Halomonas*_01 were predicted to possess this characteristic in the *H. pylori*-positive group. In contrast, among the *H. pylori*-negative markers, urease activity has been described only in some strains of *Staphylococcus aureus*. Similarly, nitrate reduction was inferred for *Pseudomonas stutzeri* and likely for *Halomonas*_01, while members of the Chitinophagaceae family have previously been associated with denitrifying activity (Zhong et al., 2017). For the *H. pylori*-negative group, this characteristic was observed in *Staphylococcus_aureus* and, with a high likelihood, in *Streptococcus*_03.

**TABLE 2:** Metabolic and physiological features of the 18 marker species differentially identified according to *H. pylori* infection status.

OPU	Species	HP status	Oxygen requirement	Gram	Urease	NO3 (Reduction of nitrates)
82	***Helicobacter pylori***	+	Microaerophile	–	+	–
1205	**Chitinophagaceae_01**	+	ND	–	ND	ND
7	**Halomonas_01**	+	Aerobe_FacAna	–	+?	+?
23	***Pseudomonas_stutzeri***	+	FacAna	–	–	+
222	***Corynebacterium kroppenstedtii***	–	FacAna	+	–	–
149	Streptococcus_03	–	FacAna	+	–	+?
270	**Bathyarchaeia_01**	–	ND	Archaea	ND	ND
238	Rathayibacter_01	–	Aerobe	+	–	–
72	***Brevundimonas vesicularis***	–	Aerobe	–	–	–
1056	Cutibacterium_02	–	ND	+	–	ND
102	***Treponema putidum***	–	ObligAna	–	ND	ND
29	*Pseudomonas formosensis*	–	Aerobe	–	ND	–
162	*Staphylococcus_aureus*	–	FacAna	+	V	+
90	Dysgonomonadaceae_01	–	ND	–	ND	ND
46	Neisseriaceae_01	–	ND	–	ND	ND
3	Haemophilus_01	–	ND	–	ND	ND
248	Absconditabacteriales_(SR1)_01	–	ND		ND	ND
226	Lawsonella_01	–	ObligAna	+	ND	ND

HP status, *H*. *pylori* status of infection; ND, not determined; V, variable. The marker species for severity in Figure 6C are highlighted in bold.

**FIGURE 9:**
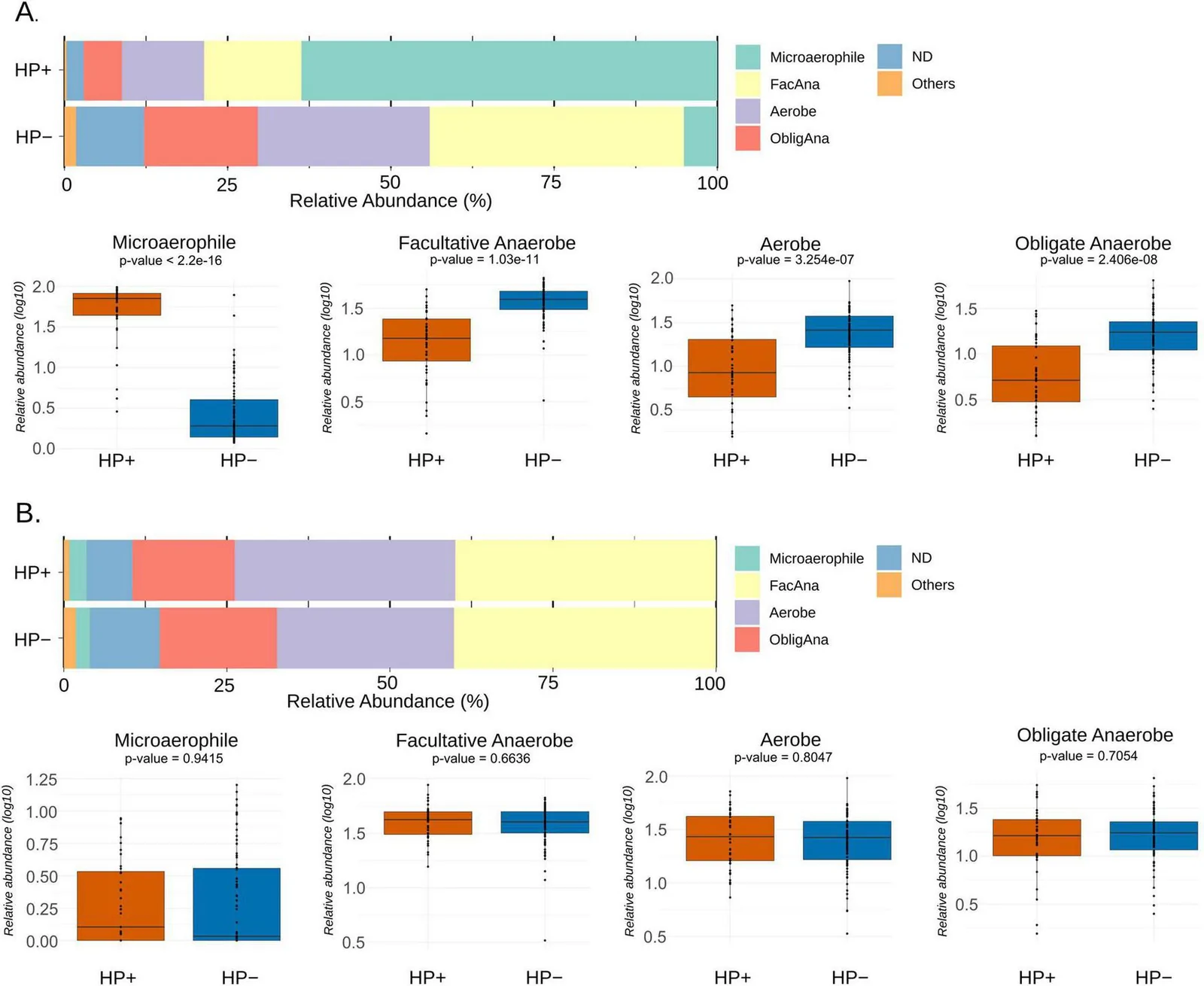
Microbial oxygen requirements according to *H. pylori* infection status. **(A)** Relative abundances (%) of species classified according to their oxygen requirements. **(B)** Comparison of the relative abundances (log10) of oxygen requirement categories between *H. pylori-positive* and *H. pylori*-negative groups. Differences between groups were assessed using the Wilcoxon rank-sum test (*p* < 0.05). HP^+^: *H. pylori*-positive group; HP^–^: *H. pylori*-negative group.

To further explore potential ecological differences between groups, microbial oxygen requirements were inferred for the mucosa-associated gastric microbiota. Fifty-five of the 293 OPUs (18.8%) could not be assigned an oxygen requirement because no reliable information was available in the reference databases, including seven of the 18 marker taxa (Table 2). With this caveat, the *H. pylori-positive* group showed a higher relative abundance of predicted microaerophiles and a lower abundance of facultative anaerobes, aerobes and obligate anaerobes than the *H. pylori*-negative group (p < 0.005) (Figure 9). However, after removing *H. pylori* reads from the dataset, these differences were no longer observed, indicating that the predominance of *H. pylori* largely accounted for the inferred physiological differences between groups.

## 4 Discussion

In this series of symptomatic children and adolescents with no peptic ulcer disease, we report a dysbiotic gastric microbiota pattern among *H. pylori-*infected subjects compared to non-infected individuals. This pattern was significantly associated with a higher score of histological gastritis but not with age or pubertal stage. The above suggests that this abnormal microbial ecosystem can be established since early life and is not harmless. Few studies have explored the interaction of *H. pylori* with other components of the gastric microbiota in children and adolescents. To our knowledge, this is the first study focusing on young individuals without peptic ulcer disease compared to non-infected subjects. This is a particularly relevant group in which eradication treatment is not currently indicated according to international pediatric guidelines (Jones et al., 2017), despite the occurrence of histological inflammation and systemic reactions (George et al., 2020; Lucero et al., 2021a; Lucero et al., 2021b; Serrano et al., 2017), including possible activation of oncogenic pathways, as it has been reported (George et al., 2020; Orellana-Manzano et al., 2016). The independence of these findings with chronological and biological age should lead to a reflection on our current pediatric guidelines. The stringent age criteria of 18 years of age to recommend eradication treatment in patients with no peptic ulcer disease lack evidence from this perspective. This recommendation is based on the described stimulatory effect of *H. pylori* on the regulatory T-cell pathway, which has a theoretically beneficial effect. It does not consider dysbiosis and the degree of histological inflammation. Since dysbiosis has been associated with a dysregulation of the Treg/Th17 balance (Calvo-Barreiro et al., 2023; Prasad et al., 2024; Strzêpa et al., 2024) and considering that this dysbiosis can also play a detrimental role in a balanced CD4 T cell Th-differentiation (Bhutta et al., 2024; Martini et al., 2023; Prasad et al., 2024), the theoretical beneficial effect of Treg improvement in infected children seems to be an oversimplification that can potentially mask immunological adverse effects. This may be particularly relevant in countries with a high prevalence of *H. pylori* infection and gastric cancer, where evidence of early gastric dysbiosis and more severe histological gastritis in symptomatic children and adolescents could support reconsideration of a more proactive approach to *H. pylori* eradication. However, such an approach should also acknowledge that eradication therapy requires combination antibiotic treatment, which may transiently perturb both the gastric and intestinal microbiota. This concern has been one of the main arguments supporting a conservative approach to eradication in children without peptic ulcer disease. Nevertheless, accumulating evidence indicates that these antimicrobial-induced alterations are largely transient, whereas *H. pylori* infection represents a chronic exposure capable of sustaining gastric dysbiosis and mucosal inflammation for decades if left untreated. In fact, several studies have demonstrated that, following successful eradication, the gastric microbiota progressively shifts toward a composition resembling that of *H. pylori*-negative individuals, with recovery of microbial diversity and community structure over time (Guo et al., 2022; He et al., 2019; Li et al., 2017; Serrano et al., 2019; Weng et al., 2021). Therefore, although the short-term ecological impact of eradication therapy should not be overlooked, it should be weighed against the sustained biological consequences of persistent *H. pylori* infection, particularly in children and adolescents living in regions with a high burden of gastric cancer (He et al., 2019).

Mucosal gastric microbiota differed in diversity and structure between *H. pylori-positive* and *H. pylori-negative* subjects. This gastric dysbiosis of *H. pylori-*infected individuals could result from local pH alterations, an inflammatory mucosal environment, and competition for adhesion sites on the gastric epithelium, among other factors (Huang et al., 2023). Our results demonstrate significant differences in the alpha diversity indices between *H. pylori-positive* and *H. pylori*-negative individuals, with higher dominance of *H. pylori* and lower heterogeneity in the *H. pylori-positive* group, consistent with previous studies in adults (Castaño-Rodríguez et al., 2017; Miftahussurur et al., 2020; Wang et al., 2022; Yu et al., 2023) and children with peptic ulcer disease (Brawner et al., 2017; Llorca et al., 2017; Zheng et al., 2021). The beta diversity analysis revealed distinct microbiota profiles between *H. pylori-positive* and *H. pylori*-negative groups, suggesting that the presence of *H. pylori* could significantly influence the overall composition of the gastric microbiota.

According to Random Forest analysis, there were at least three taxa consistently associated with the presence of *H. pylori* and histological gastritis, suggesting that these bacteria are representatives of the altered status of the gastric microbial community. Previous reports have also found these organisms to be associated with *H. pylori*-related pathologies and even in the context of gastric cancer. Liu et al. (2019) identified *Halomonas* as one of the most abundant genera in peritumoral gastric tissue, and Ferreira et al. (2018) found Chitinophagaceae as one of the enriched families in gastric microbiota of patients with chronic gastritis. *Pseudomonas stutzeri* is also part of the gastric microbiota, but results are variable. Jo et al. (2016) found it in gastric biopsies of both *H. pylori*-negative individuals and *H. pylori-positive* patients with gastric cancer. Although the abundance (mean proportion) was higher in the first group, the difference was non-significant. In contrast, Wang et al. found *Pseudomonas stutzeri* as one of the species with a significantly higher abundance in gastric biopsies from *H. pylori*-negative individuals compared to those obtained from *H. pylori-positive* individuals (Wang et al., 2022).

The most abundant microbial phyla in gastric microbiota from *H. pylori*-negative patients were those previously described as normal inhabitants in gastric mucosa biopsies, i.e., Firmicutes, Proteobacteria, Actinobacteria, and Bacteroidota. Among Firmicutes, *Streptococcus* was identified as a genus marker for the *H. pylori*-negative group and as the only bacterial taxa found in a significant negative correlation with *H. pylori* abundance. This observation is consistent with previous data indicating that *Streptococcus* is one of the dominant taxa in the gastric microbiota of *H. pylori*-negative individuals (Schulz et al., 2018), even in samples of individuals with gastric cancer (Jo et al., 2016). On the other hand, the case of the archaea class Bathyarchaeia was noticeable, which was found to be a marker taxon of the *H. pylori*-negative group, with a significant negative correlation with the *H. pylori* abundance. Bathyarchaea are recognized as one of the most abundant microbial groups on Earth (Miftahussurur et al., 2020), but to our knowledge, there are no previous reports of their presence in the gastric microbiota. More research is required to elucidate the potential role of archaea species within the gastric microhabitat.

Diversity is presumed to contribute to a greater stability of the host microbiome, making it resilient to the effects of acute infections, antibiotics, and immunosuppression (Safarchi et al., 2025). Gastrointestinal dysbiosis in children has been associated with many metabolic, cardiovascular, and even neurological pathologies (Lupu et al., 2026a; Lupu et al., 2026b; Saeed et al., 2022; Zhang et al., 2026). Given that several of these disorders predominantly manifest during adolescence or adulthood (Sarkar et al., 2021), their long-term impact could potentially be reduced by preventing or limiting *H. pylori*-associated gastric dysbiosis during childhood. Previous data indicated that the altered gastric microbiota of *H. pylori-positive* individuals may change to a status similar to that found in *H. pylori*-negative individuals several months after eradication (Guo et al., 2022; Li et al., 2017; Serrano et al., 2019).

We also explored the association of gastric microbiota, including *H. pylori*, with the presence and severity of histological gastritis, which has been considered in only a few pediatric studies (Brawner et al., 2017; Llorca et al., 2017; Zheng et al., 2021; Zheng et al., 2024). The composition of the gastric microbial communities differed between the *H. pylori-positive* group with gastritis and the *H. pylori*-negative groups, irrespective of the presence of gastritis. In addition, the severity of histological gastritis was significantly correlated with the presence of *H. pylori* and the three other coexisting taxa, consistent with the findings of Zheng et al. in Chinese children (Zheng et al., 2021). Brawner et al. also reported a distinct gastric microbiota profile between symptomatic children with and without *H. pylori* infection and higher expression of certain Treg markers in infected patients (Brawner et al., 2017). However, they did not analyze the severity of histological gastritis, and immune-mediated gastrointestinal diseases were not excluded, raising the possibility that the lower Treg expression observed in uninfected individuals reflected underlying inflammatory conditions rather than true absence of *H. pylori*. In contrast, our study excluded patients with any immune-mediated disease, thereby minimizing this potential confounding factor.

This finding was independent of gender, chronological, and biological age of the subjects, suggesting that factors other than age and gender may play a more significant role in shaping the gastric microbiota in pediatric patients. It also supports the reduction of the threshold for eradication treatment to earlier ages, considering the dysbiosis and histological gastritis associated with *H. pylori*, as previously stated.

To gain insight into the potential functional characteristics of the bacterial consortium, we explored the distribution of taxonomically inferred metabolic and physiological traits. It can be observed that urease and nitrate reduction activity were the most common characteristics in the *H. pylori-*positive group. These traits have previously been associated with *H. pylori* colonization, hypochlorhydria, and increased intragastric nitrite production. Although these mechanisms were not evaluated in the present study, they provide a plausible biological context for the more severe histological gastritis observed in *H. pylori-*positive patients (Ojima et al., 2022; Song C. et al., 2026; Wang et al., 2026).

This study has several limitations that should be considered when interpreting our findings. First, its cross-sectional design precludes establishing causal relationships between *H. pylori* infection, gastric dysbiosis, and histological gastritis, and longitudinal studies will be required to determine the temporal sequence of these events and the impact of eradication on restoration of gastric eubiosis. Second, the gastric microbiota was characterized from mucosal biopsies obtained from the antrum, which may not fully represent the microbial communities across the entire stomach. Third, our cohort consisted exclusively of symptomatic Chilean children and adolescents undergoing upper gastrointestinal endoscopy, and therefore, the findings may not be directly generalizable to asymptomatic populations or to regions with different epidemiological backgrounds. In addition, the analyses of *cagA* and *vacA* genotypes were limited by the relatively small number of *H. pylori*-positive subjects, particularly in some *vacA* subgroups, reducing statistical power to detect modest associations. Finally, the metabolic and physiological characteristics assigned to bacterial taxa were inferred from curated reference databases and published species descriptions rather than experimentally measured in this cohort; therefore, these should be interpreted as putative functional traits rather than confirmed microbial phenotypes. Nevertheless, despite these limitations, the prospective multicenter design, the rigorous definition of *H. pylori* infection using three complementary diagnostic methods, the integration of detailed histological assessment with mucosa-associated microbiota profiling, and the inclusion of a clinically relevant but poorly studied pediatric population provide robust evidence that *H. pylori* infection is associated with early gastric dysbiosis and increased mucosal inflammation. These findings establish an important foundation for future longitudinal and mechanistic studies aimed at defining whether earlier eradication strategies may improve long-term gastric health in children and adolescents from regions with a high burden of gastric cancer.

In conclusion, our study provides new insights into the composition and diversity of the gastric microbiota in pediatric patients with *H. pylori* infection and gastritis without peptic ulcer disease. *H. pylori* infection and histological gastritis were associated with specific changes in the gastric microbiota composition, highlighting the complex interplay between host, microbiota, and disease. Moreover, our findings suggest that *H. pylori*-associated dysbiosis occurs independently of chronological or biological age. These findings support reconsideration of a more proactive approach to *H. pylori* eradication at earlier stages, even in the absence of erosive or ulcerative lesions, with the aim of limiting *H. pylori*-associated gastric dysbiosis and potentially reducing the risk of long-term gastric complications.

## Data Availability

The datasets presented in this study can be found in online repositories. The names of the repository/repositories and accession number(s) can be found at: https://www.ncbi.nlm.nih.gov/, PRJNA1257435.

## Footnotes

1
www.mrdnalab.com

2
https://github.com/nowthis/sankeymatic
