# Supplementary material for: Gastric dysbiosis and more severe histological gastritis in symptomatic Helicobacter pylori-infected children and adolescents without ulcers: time to rethink early eradication?
Source: Front Microbiol. 2026 Aug 28;17:1927606. doi: 10.3389/fmicb.2026.1927606 (PMC13564255; doi:10.3389/fmicb.2026.1927606)
Supplement: Supplementary file 1 [file Table_1.docx]

Supplementary Tables

Table S1. Alpha diversity indices by patient according to *H. pylori* infection status.

| Sample | *H. pylori* infection | Nr_Reads | Nr_OPUs | Chao-1 | Dominance_D | Shannon_H | Evenness_e^H/S |
| --- | --- | --- | --- | --- | --- | --- | --- |
| 1 | Negative | 18807 | 51 | 51 | 0.0897 | 2.841 | 0.3361 |
| 2 | Positive | 59989 | 50 | 50 | 0.4147 | 1.556 | 0.09475 |
| 3 | Positive | 24463 | 45 | 45 | 0.1966 | 2.352 | 0.2335 |
| 4 | Negative | 20276 | 48 | 48 | 0.1213 | 2.662 | 0.2985 |
| 5 | Negative | 28573 | 43 | 43 | 0.07564 | 2.858 | 0.4054 |
| 6 | Positive | 40003 | 44 | 44 | 0.1751 | 2.443 | 0.2615 |
| 7 | Negative | 45189 | 46 | 46 | 0.09507 | 2.65 | 0.3078 |
| 8 | Positive | 53152 | 38 | 38 | 0.2737 | 1.983 | 0.1912 |
| 9 | Negative | 16165 | 33 | 33 | 0.1097 | 2.502 | 0.3698 |
| 10 | Negative | 21070 | 47 | 47 | 0.1578 | 2.376 | 0.2289 |
| 11 | Negative | 41804 | 45 | 45 | 0.08217 | 2.766 | 0.3532 |
| 12 | Positive | 48290 | 65 | 65 | 0.681 | 1.005 | 0.04203 |
| 13 | Negative | 37304 | 90 | 90 | 0.1178 | 3.171 | 0.2647 |
| 14 | Positive | 43196 | 58 | 58 | 0.08765 | 2.965 | 0.3343 |
| 15 | Positive | 9367 | 36 | 36 | 0.1297 | 2.61 | 0.3776 |
| 16 | Negative | 16254 | 43 | 43 | 0.07383 | 2.832 | 0.395 |
| 17 | Negative | 25057 | 46 | 46 | 0.06254 | 3.024 | 0.4471 |
| 18 | Negative | 13588 | 47 | 47 | 0.06523 | 3.048 | 0.4483 |
| 19 | Positive | 41635 | 47 | 47 | 0.4684 | 1.514 | 0.09668 |
| 20 | Negative | 53719 | 57 | 57 | 0.1332 | 2.396 | 0.1925 |
| 21 | Positive | 51516 | 36 | 36 | 0.6186 | 0.9171 | 0.0695 |
| 22 | Positive | 46167 | 47 | 47 | 0.5404 | 1.333 | 0.08066 |
| 23 | Positive | 26734 | 45 | 45 | 0.2697 | 2.064 | 0.175 |
| 24 | Negative | 11960 | 36 | 36 | 0.1016 | 2.667 | 0.3998 |
| 25 | Negative | 21215 | 66 | 66 | 0.07323 | 3.085 | 0.3313 |
| 26 | Negative | 36307 | 43 | 43 | 0.05734 | 3.099 | 0.5158 |
| 27 | Negative | 40237 | 56 | 56 | 0.5559 | 1.4 | 0.0724 |
| 28 | Positive | 37479 | 48 | 48 | 0.5082 | 1.359 | 0.08109 |
| 29 | Positive | 20794 | 58 | 58 | 0.0775 | 2.98 | 0.3393 |
| 30 | Negative | 21276 | 43 | 43 | 0.07763 | 2.791 | 0.3792 |
| 31 | Negative | 10617 | 39 | 39 | 0.07317 | 2.888 | 0.4603 |
| 32 | Negative | 18573 | 32 | 32 | 0.08366 | 2.715 | 0.472 |
| 33 | Negative | 14361 | 46 | 46 | 0.1054 | 2.71 | 0.3268 |
| 34 | Negative | 19710 | 41 | 41 | 0.08057 | 2.809 | 0.4049 |
| 35 | Negative | 14759 | 44 | 44 | 0.08515 | 2.747 | 0.3546 |
| 36 | Positive | 24724 | 42 | 42 | 0.2408 | 2.268 | 0.2299 |
| 37 | Negative | 16413 | 43 | 43 | 0.1286 | 2.655 | 0.3308 |
| 38 | Negative | 10766 | 74 | 74 | 0.05247 | 3.483 | 0.4401 |
| 39 | Positive | 13398 | 43 | 43 | 0.1299 | 2.532 | 0.2925 |
| 40 | Positive | 22219 | 25 | 25 | 0.7399 | 0.7889 | 0.08804 |
| 41 | Positive | 34077 | 47 | 47 | 0.1002 | 2.794 | 0.3477 |
| 42 | Negative | 31032 | 43 | 43 | 0.08901 | 2.876 | 0.4125 |
| 43 | Positive | 12619 | 39 | 39 | 0.1321 | 2.474 | 0.3044 |
| 44 | Negative | 36405 | 44 | 44 | 0.08169 | 2.76 | 0.3591 |
| 45 | Negative | 12018 | 43 | 43 | 0.06023 | 3.123 | 0.5282 |
| 46 | Negative | 8316 | 42 | 42 | 0.07013 | 2.933 | 0.4471 |
| 47 | Negative | 3241 | 47 | 47 | 0.04001 | 3.465 | 0.6805 |
| 48 | Negative | 18859 | 44 | 44 | 0.07533 | 2.877 | 0.4037 |
| 49 | Negative | 6771 | 47 | 47 | 0.04884 | 3.295 | 0.5741 |
| 50 | Negative | 17605 | 65 | 65 | 0.06228 | 3.252 | 0.3975 |
| 51 | Negative | 16985 | 52 | 52 | 0.05081 | 3.266 | 0.5041 |
| 52 | Negative | 30000 | 53 | 53 | 0.07056 | 2.97 | 0.3677 |
| 53 | Negative | 29602 | 58 | 58 | 0.05984 | 3.088 | 0.3783 |
| 54 | Negative | 30032 | 52 | 52 | 0.06573 | 3.003 | 0.3874 |
| 55 | Positive | 50131 | 88 | 88 | 0.2239 | 2.585 | 0.1507 |
| 56 | Negative | 27091 | 92 | 92.5 | 0.05645 | 3.468 | 0.3486 |
| 57 | Negative | 21541 | 43 | 43 | 0.09522 | 2.677 | 0.3381 |
| 58 | Negative | 14276 | 46 | 46 | 0.1024 | 2.704 | 0.3247 |
| 59 | Positive | 35955 | 23 | 23 | 0.9363 | 0.232 | 0.05483 |
| 60 | Negative | 31980 | 42 | 42 | 0.0851 | 2.79 | 0.3878 |
| 61 | Negative | 26783 | 44 | 44 | 0.1424 | 2.252 | 0.216 |
| 62 | Positive | 22097 | 43 | 43 | 0.09174 | 2.696 | 0.3446 |
| 63 | Negative | 37602 | 63 | 63 | 0.1743 | 2.499 | 0.1931 |
| 64 | Negative | 25197 | 40 | 40 | 0.09099 | 2.69 | 0.3684 |
| 65 | Negative | 16654 | 36 | 36 | 0.1061 | 2.494 | 0.3363 |
| 66 | Negative | 35839 | 54 | 54 | 0.1161 | 2.53 | 0.2325 |
| 67 | Negative | 28136 | 40 | 40 | 0.1269 | 2.436 | 0.2857 |
| 68 | Negative | 18535 | 32 | 32 | 0.1141 | 2.378 | 0.3369 |
| 69 | Positive | 36400 | 43 | 43 | 0.1886 | 2.246 | 0.2198 |
| 70 | Negative | 25510 | 51 | 51 | 0.08683 | 2.761 | 0.3102 |
| 71 | Negative | 31177 | 37 | 37 | 0.1097 | 2.556 | 0.3481 |
| 72 | Negative | 23693 | 57 | 57 | 0.06951 | 3.052 | 0.3712 |
| 73 | Positive | 42626 | 41 | 41 | 0.6568 | 0.9996 | 0.06627 |
| 74 | Negative | 21603 | 39 | 39 | 0.09927 | 2.634 | 0.357 |
| 75 | Positive | 21353 | 32 | 32 | 0.5267 | 1.32 | 0.117 |
| 76 | Negative | 23430 | 42 | 42 | 0.2175 | 2.175 | 0.2095 |
| 77 | Negative | 22172 | 30 | 30 | 0.1346 | 2.315 | 0.3374 |
| 78 | Positive | 31900 | 34 | 34 | 0.4744 | 1.434 | 0.1234 |
| 79 | Positive | 55406 | 51 | 51 | 0.797 | 0.6532 | 0.03768 |
| 80 | Positive | 27237 | 41 | 41 | 0.2775 | 1.909 | 0.1645 |
| 81 | Negative | 41868 | 69 | 69 | 0.05695 | 3.24 | 0.3702 |
| 82 | Negative | 19982 | 67 | 67 | 0.05071 | 3.317 | 0.4117 |
| 83 | Negative | 13354 | 48 | 48 | 0.07203 | 2.988 | 0.4135 |
| 84 | Negative | 18227 | 56 | 56 | 0.09966 | 2.706 | 0.2673 |
| 85 | Positive | 31829 | 33 | 33 | 0.6583 | 0.9666 | 0.07967 |
| 86 | Negative | 29952 | 53 | 53 | 0.1017 | 2.622 | 0.2597 |
| 87 | Negative | 32738 | 56 | 56 | 0.08684 | 2.893 | 0.3222 |
| 88 | Negative | 26601 | 33 | 33 | 0.1383 | 2.286 | 0.2982 |
| 89 | Positive | 42676 | 30 | 30 | 0.817 | 0.558 | 0.05824 |
| 90 | Negative | 33481 | 70 | 70 | 0.08086 | 2.884 | 0.2556 |
| 91 | Negative | 13488 | 58 | 58 | 0.09555 | 2.954 | 0.3308 |
| 92 | Negative | 12002 | 46 | 46 | 0.1069 | 2.657 | 0.3097 |
| 93 | Negative | 30557 | 41 | 41 | 0.07923 | 2.862 | 0.4267 |
| 94 | Negative | 16030 | 38 | 38 | 0.1087 | 2.611 | 0.3582 |
| 95 | Negative | 12750 | 43 | 43 | 0.06176 | 3.024 | 0.4783 |
| 96 | Positive | 35712 | 42 | 42 | 0.599 | 1.158 | 0.07584 |
| 97 | Negative | 31804 | 59 | 59 | 0.07544 | 3.002 | 0.3412 |
| 98 | Negative | 18872 | 47 | 47 | 0.1935 | 2.427 | 0.2411 |
| 99 | Negative | 9897 | 38 | 38 | 0.1343 | 2.466 | 0.3099 |
| 100 | Negative | 23464 | 44 | 44 | 0.2709 | 1.941 | 0.1583 |
| 101 | Negative | 42734 | 69 | 69 | 0.05771 | 3.217 | 0.3616 |
| 103 | Negative | 4185 | 42 | 42 | 0.08676 | 3.01 | 0.4832 |
| 104 | Negative | 31518 | 40 | 40 | 0.8442 | 0.55 | 0.04333 |
| 105 | Negative | 7350 | 51 | 51 | 0.06377 | 3.279 | 0.5205 |
| 106 | Positive | 27067 | 37 | 37 | 0.7508 | 0.8046 | 0.06043 |
| 107 | Negative | 4123 | 41 | 41 | 0.07255 | 3.117 | 0.5506 |
| 108 | Negative | 12233 | 71 | 71 | 0.04383 | 3.635 | 0.5341 |
| 109 | Positive | 12430 | 52 | 52 | 0.281 | 2.316 | 0.195 |
| 110 | Positive | 24070 | 32 | 32 | 0.7883 | 0.6568 | 0.06027 |
| 111 | Positive | 11168 | 38 | 38 | 0.594 | 1.238 | 0.09072 |
| 112 | Positive | 17912 | 18 | 18 | 0.8978 | 0.3376 | 0.07786 |
| 113 | Positive | 15010 | 36 | 36 | 0.6615 | 1.038 | 0.07847 |
| 114 | Positive | 24790 | 52 | 52 | 0.5944 | 1.284 | 0.06941 |
| 115 | Positive | 33834 | 23 | 23 | 0.9398 | 0.2171 | 0.05402 |
| 116 | Positive | 11002 | 52 | 52 | 0.2008 | 2.567 | 0.2504 |
| 117 | Negative | 7652 | 59 | 59 | 0.1109 | 2.926 | 0.316 |
| 118 | Negative | 7474 | 64 | 64 | 0.1039 | 2.989 | 0.3103 |
| 119 | Positive | 14759 | 46 | 46 | 0.452 | 1.699 | 0.1188 |

**Table S2.** Microbiological taxa identified as significantly different according to *H. pylori* infection status.

| **tax_name** | **log2_median_ratio** | **wilcox_p_value** | **q_FDR** |
| --- | --- | --- | --- |
| Helicobacter_pylori | 7,477345723 | 4.40E-16 | **1.29E-13** |
| Corynebacterium_kroppenstedtii | -1,404333998 | 8.04E-09 | **1.18E-06** |
| Chitinophagaceae_01 | 0 | 4.55E-06 | **4.44E-04** |
| Bathyarchaeia_01 | ´=-Inf | 1.90E-05 | **1.21E-03** |
| Cutibacterium_02 | -2,472825893 | 2.34E-05 | **1.21E-03** |
| Halomonas_01 | 0 | 2.48E-05 | **1.21E-03** |
| Streptococcus_03 | -2,098160951 | 4.02E-05 | **1.60E-03** |
| Treponema_putidum | -2,405650443 | 4.38E-05 | **1.60E-03** |
| Brevundimonas_vesicularis | -1,449725013 | 1.04E-04 | **3.39E-03** |
| Rathayibacter_01 | -2,606338536 | 1.98E-04 | **5.79E-03** |
| Pseudomonas_formosensis | -2,384208958 | 6.83E-04 | **0.0182** |
| Pseudomonas_stutzeri | 0 | 1.45E-03 | **0.0351** |
| Pseudomonas_01 | ¨=-Inf | 1.56E-03 | **0.0351** |
| Dysgonomonadaceae_01 | -2,624249176 | 1.89E-03 | **0.0396** |
| Staphylococcus_aureus | -2,232759479 | 3.17E-03 | 0.0592 |
| Absconditabacteriales_(SR1)_01 | #¿Name? | 3.23E-03 | 0.0592 |
| Novosphingobium_01 | 0 | 4.81E-03 | 0.0828 |
| Paludibacter_01 | 0 | 5.14E-03 | 0.0828 |
| Flavobacteriales_NS9_marine_group_01 | 0 | 5.37E-03 | 0.0828 |
| Haemophilus_01 | 0 | 6.53E-03 | 0.0956 |
| Corynebacterium_01 | -2,198347191 | 7.20E-03 | 0.1 |
| Firmicutes_Incertae_Sedis_DTU014_01 | 0 | 8.27E-03 | 0.11 |
| Akkermansia_01 | 0 | 8.78E-03 | 0.112 |
| Pirellulaceae_Pir4_lineage_01 | 0 | 9.44E-03 | 0.113 |
| Neisseriaceae_01 | -0,814798097 | 9.68E-03 | 0.113 |
| Acinetobacter_03 | 0 | 0.0124 | 0.14 |
| Actinomyces_01 | 0 | 0.0134 | 0.145 |
| Prevotellaceae_UCG_003_01 | 0 | 0.0151 | 0.159 |
| Rhizobium_subbaraonis | 0 | 0.0168 | 0.169 |
| Blautia_01 | 0 | 0.0202 | 0.197 |
| Brachyspirales_LH041_01 | 0 | 0.0212 | 0.197 |
| Blautia_02 | -4,498081185 | 0.0216 | 0.197 |
| Sphingomonas_desiccabilis | 0 | 0.0239 | 0.213 |
| Lachnoanaerobaculum_orale | 0 | 0.0257 | 0.221 |
| Neisseria_oralis | ´=-Inf | 0.0271 | 0.222 |
| Staphylococcus_05 | 0 | 0.0273 | 0.222 |
| Bacteroidales_Bacteroidetes_BD2_2_01 | 0 | 0.0297 | 0.235 |
| Streptococcus_04 | 0 | 0.0308 | 0.238 |
| Afipia_01 | 0 | 0.0338 | 0.251 |
| Dehalobacter_01 | 0 | 0.0343 | 0.251 |
| Micrarchaeales_01 | 0 | 0.0375 | 0.259 |
| Saprospiraceae_01 | 0 | 0.0376 | 0.259 |
| Prolinoborus_fasciculus | -1,323348032 | 0.0382 | 0.259 |
| Pseudomonas_mediterranea | 0 | 0.0389 | 0.259 |
| Methylobacterium_dankookense | 0 | 0.0437 | 0.285 |
| Corynebacterium_vitaeruminis | 0 | 0.0488 | 0.304 |
| Kocuria_02 | 0 | 0.0488 | 0.304 |

**Table S3.** Correlations of Figure 4.

| Taxon1 | Taxon2 | Correlation | P.Value |
| --- | --- | --- | --- |
| Cutibacterium_02 | Pseudomonas_formosensis | 0.6849 | 0.0099 |
| Brevundimonas_vesicularis | Pseudomonas_formosensis | 0.5968 | 0.0099 |
| Pseudomonas_formosensis | Pseudomonas_stutzeri | **-0.5865** | 0.0099 |
| Brevundimonas_vesicularis | Cutibacterium_02 | 0.5233 | 0.0099 |
| Cutibacterium_02 | Pseudomonas_stutzeri | **-0.5182** | 0.0099 |
| Chitinophagaceae_01 | Halomonas_01 | 0.5127 | 0.0099 |
| Halomonas_01 | Helicobacter_pylori | 0.4821 | 0.0099 |
| Chitinophagaceae_01 | Helicobacter_pylori | 0.458 | 0.0099 |
| Cutibacterium_02 | Streptococcus_03 | 0.4325 | 0.0099 |
| Dysgonomonadaceae_01 | Rathayibacter_01 | 0.4255 | 0.0099 |
| Pseudomonas_formosensis | Streptococcus_03 | 0.3994 | 0.0099 |
| Bathyarchaeia_01 | Pseudomonas_stutzeri | **-0.3716** | 0.0099 |
| Brevundimonas_vesicularis | Pseudomonas_stutzeri | **-0.3711** | 0.0099 |
| Helicobacter_pylori | Streptococcus_03 | **-0.3511** | 0.0099 |
| Pseudomonas_stutzeri | Streptococcus_03 | **-0.3466** | 0.0099 |
| Bathyarchaeia_01 | Helicobacter_pylori | **-0.3463** | 0.0099 |
| Corynebacterium_kroppenstedtii | Staphylococcus_aureus | 0.3238 | 0.0099 |
| Brevundimonas_vesicularis | Streptococcus_03 | 0.312 | 0.0198 |
